# Survival analysis of a stochastic impulsive single-species population model with migration driven by environmental toxicant

**DOI:** 10.1038/s41598-023-37861-z

**Published:** 2023-07-03

**Authors:** Xiangjun Dai, Jianjun Jiao, Qi Quan

**Affiliations:** 1 School of Mathematical Sciences, Guizhou Normal University, Guiyang, 550025 People’s Republic of China; 2 School of Date science, Tongren University, Tongren, 554300 People’s Republic of China; 3 School of Mathematics and Statistics, Guizhou University of Finance and Economics, Guiyang, 550025 People’s Republic of China

**Keywords:** Dynamical systems, Applied mathematics, Stochastic modelling

## Abstract

Considering the influence of environmental toxicant on population migration between patches, we propose and study a stochastic impulsive single-species population model with migration driven by environmental toxicant in this paper. We first discuss the existence and uniqueness of global positive solutions of the model by constructing the Lyapunov function. Then, we obtain sufficient conditions for extinction, stochastic persistence and persistence in the mean of the single-species population. Finally, we present some numerical simulations to illustrate our results. These results provide insights for the conservation and management of species in polluted environments.

## Introduction

Due to differences in the geographical environment and the influence of human activities, the habitats of many species are broken up into isolated patches, which may lead to the extinction of species within the patch. Therefore, the study of population migration between patches plays a very important role in the conservation and management of species, and many scholars have analysed the effects of migration on stability, permanence, extinction, and other dynamic properties by establishing mathematical models (see^1–14^). For example, Feng et al.^9^ proposed and studied a predator-prey model with predator population migration dependent on prey. Kang et al.^10^ considered the situation that predators migrate towards patches with more concentrated predator-prey interactions in the model. Specifically, some scholars proposed single-species population models with migrations between the non-nature reserve and the nature reserve to study the survival and extinction of single-species populations. For example, Zou and Wang^11^ proposed and studied the following deterministic single-species diffusion model.

$$\begin{aligned} \left\{ \begin{aligned} \frac{dx_1(t)}{dt}&=x_1(t)(r-ax_1(t))+\frac{D}{H}(x_2(t)-x_1(t)),\\ \frac{dx_2(t)}{dt}&=x_2(t)(r-ax_2(t))+\frac{D}{h}(x_1(t)-x_2(t)), \end{aligned}\right. \end{aligned}$$

where $r>0$ and $a>0$ stand for the population growth rate and the intra-specific competition coefficient of population. $D>0$ is the diffusion coefficient. *H* and *h* are sizes of the non-nature reserve and the nature reserve. And then, the extinction and permanence in the mean of single species under fluctuated environments were also studied by Zou et al.^12,13^ and Dieu et al.^14^. Based on the model in^12^, Wei and Wang^15^ established the following stochastic single-species model with migrations between two patches.

$$\begin{aligned} \left\{ \begin{aligned} dx_1(t)&=\left[ x_1(t)(r-ax_1(t))+(d_{21}x_2(t)-d_{12}x_1(t))-E_1x_1(t)\right] dt+\sigma x_1(t)dB(t),\\ dx_2(t)&=\left[ x_2(t)(r-ax_2(t))+(d_{12}x_1(t)-d_{21}x_2(t))-E_2x_2(t)\right] dt+\sigma x_2(t)dB(t), \end{aligned}\right. \end{aligned}$$

where $d_{12}\ge 0$ stands for the migration rate of the population from the non-nature reserve (patch 1) to the nature reserve (patch 2), $d_{21}\ge 0$ stands for the migration rate of the population from the nature reserve to the non-nature reserve. $E_i$ denotes the hunting rate in the *i*-th patch, and $E_1\gg E_2$. *B*(*t*) is standard Brownian motion. In^15^, authors assumed that the number of individuals of a species in the nature reserve is larger than that in the non-nature reserve, and sufficient conditions for the extinction and persistence in the mean of population were obtained. However, it is not difficult to find that the growth rate, the intra-specific competition coefficient and the intensity of white noise in two patches are the same, so the results obtained in^15^ are not suitable for the general situation. Therefore, we need to further discuss the influence of population migration on the survival of single-species.

With the rapid development of human society, a large number of toxic substances and pollutants are discharged into the ecosystem, seriously polluting the ecological environment and threatening the survival of species. Such as heavy metal pollution, and water pollution caused by crop fertilization and pesticide application. Therefore, it is most important to investigate the survival and extinction of species in a polluted environment. In recent years, many excellent results have analyzed the effects of toxicant discharged into the environment from modern industry and modern agriculture on population by establishing models^16–21^. But, these models mainly discussed the effect of pollutants on the population growth rate. As we all know, many creatures in nature have good sensory organs and highly differentiated nervous systems, and they can respond to information in the environment accordingly. For example, in agricultural production, many pests will choose to escape from the pesticide-treated environment due to the stimulation of chemical pesticides, and then seek a new environment conducive to population growth, this may be one of the reasons for inducing the resurgence of pest populations and the emergence of pest resistance. Therefore, it is necessary to consider the effect of environmental toxicant on population migration. Wei el at.^20,21^ proposed two single-species population models with physiological effect, where the “physiological” effect is described as self-protection by organisms in highly polluted environments to reduce the effective contact between the organism and the polluted environment. However, few studies have considered the influence of environmental toxicant on population migration between patches. In this paper, we assume that toxins are emitted in regular pulses, a common example being the use of pesticides, and propose a deterministic single-species population model with migration driven by environmental toxicant as follows:

1 $$\begin{aligned} \left\{ \begin{aligned}{}&\left. \begin{aligned}{}&\frac{dx_1(t)}{dt}=x_1(t)[r_{1e}-\delta c_o(t)-a_1x_1(t)]+d_{21}x_2(t)-d_{12}(1+\frac{\rho c_e^2(t)}{1+\alpha c_e^2(t)})x_1(t),\\&\frac{dx_2(t)}{dt}=x_2(t)[r_{2e}-a_2x_2(t)]+d_{12}(1+\frac{\rho c_e^2(t)}{1+\alpha c_e^2(t)})x_1(t)-d_{21}x_2(t),\\&\frac{dc_o(t)}{dt}=fc_e(t)-(g+m)c_o(t),\\&\frac{dc_e(t)}{dt}=-hc_e(t), \end{aligned}\right\} ~~t\ne n\gamma ,\\&\Delta x_1(t)=0, \Delta x_2(t)=0, \Delta c_o(t)=0, \Delta c_e(t)=b,~~ t=n\gamma ,~~ n\in Z^+. \end{aligned}\right. \end{aligned}$$

Here $x_i(t)$ denotes the density of population in patch *i*. $c_e(t)$ and $c_o(t)$ represent the concentration of toxicant in the environment and organism at time *t* respectively. $f>0$ represents the uptake rate of toxicant from the environment by the population in patch 1. $(g+m)c_o(t)$ describes loss due to egestion and metabolic process at time *t*. $b\ge 0$ and $\gamma >0$ represent the pulse input amount of toxins and the pulse input period of toxicant respectively. *hc*(*t*) represents the total lose at time *t* from the system environment including processes such as biological transformation, microbial degradation, volatilization and photosynthetic degradation. $\delta c_o(t)$ represents the lethal rate of toxins in the organism to the population in patch 1. In this paper, we adopt a Holling-III response function $\frac{\rho d_{12} c_e^2}{1+\alpha c_e^2}$ to describe the influence of toxicant concentration in patch 1 on population migration. $\rho d_{12}$ is described as the migration rate of the population in patch 1 to patch 2 due to the stimulation of toxicant in patch 1, and $\alpha >0$ denotes the sensitivity of population to environmental toxicant. $\Delta \psi (t)=\psi (t^+)-\psi (t)$ $(\psi =x_1,~x_2,~c_o,~c_e)$, $\psi (t^+)=\lim \limits _{s\rightarrow 0^+}\psi (t+s)$.

On the other hand, the population is inevitably affected by various factors in the environment, for example, changes in temperature, climate and weather. May^22^ showed that the birth rates, carrying capacity, and other parameters involved in the system can be affected by environmental noise. In order to better understand the dynamic behaviors of the population models, many researchers introduced random perturbations into deterministic models to show richer and more complex dynamic properties^24–31^. Motivated by the above studies, we suppose that environmental noises mainly affect the growth rate $r_{ie}$ of system (1) in this paper, according to the central limits theorem, we usually use an average value plus an error term satisfying the standard normal distribution to estimate a value^25,26^, that is,

$$\begin{aligned} r_{ie}(t)=r_{ie}+\sigma _i \frac{dB_i(t)}{dt},~i=1,~2., \end{aligned}$$

where $r_{ie}$ is a positive constant, $\frac{dB_i(t)}{dt}$ is the a Gaussian white noise, $B_i(t)$ represents the standard Brownian motion defined on the complete probability space $(\Omega , \mathcal {F}, \{\mathcal {F}_t\}_{t\ge 0}, \mathbb {P})$ with $\{\mathcal {F}_t\}_{t\ge 0}$ satisfying the usual conditions^23^. $\sigma _i$ is the intensity of the white noise. There is another possible form of modeling for $r_{ie}$ in a randomly-varying environment, we introduce the Ornstein-Uhlenbeck process (also called as mean-reverting process)^21–27^, and it has the following form

2 $$\begin{aligned} dr_{ie}(t)=\mu _i(r_{ie}-r_i(t))dt+\xi _i dB_i(t), \end{aligned}$$

where $r_{ie},~\xi _i$ and $\mu _i$ are positive constants, $\mu _i$ is the speed of reversion and $\xi _i$ is the intensity of the white noise. Solving the stochastic Eq. (2), from studies^21–27^, we have

$$\begin{aligned} r_{ie}(t)=r_{ie}+(r_{i0}-r_{ie})e^{-\mu _i t}+\sigma _i(t)\frac{dB_i(t)}{dt}, \end{aligned}$$

where $r_{i0}=r_i(0)$ and $\sigma _i(t)=\frac{\xi _i}{\sqrt{2\mu _i}}\sqrt{1-e^{-2\mu _i t}}$. Modifying the deterministic model (1), we propose the following stochastic impulsive single-species population model with migration driven by environmental toxicant

3 $$\begin{aligned} \left\{ \begin{aligned}{}&\left. \begin{aligned}{}&dx_1(t)=x_1(t)[r_{1e}+(r_{10}-r_{1e})e^{-\mu _1 t}-\delta c_o(t)-a_1x_1(t)]dt+[d_{21}x_2(t)\\&~~~~~~~~-d_{12}(1+\frac{\rho c_e^2(t)}{1+\alpha c_e^2(t)})x_1(t)]dt+\sigma _1(t)x_1(t)dB_1(t),\\&dx_2(t)=x_2(t)[r_{2e}+(r_{20}-r_{2e})e^{-\mu _2 t}-a_2x_2(t)]dt+[d_{12}(1+\frac{\rho c_e^2(t)}{1+\alpha c_e^2(t)})x_1(t),\\&~~~~~~~~-d_{21}x_2(t)]dt+\sigma _2(t)x_2(t)dB_2(t),\\&dc_o(t)=fc_e(t)-(g+m)c_o(t) dt,\\&dc_e(t)=-hc_e(t)dt, \end{aligned}\right\} ~~t\ne n\gamma ,\\&\Delta x_1(t)=0, \Delta x_2(t)=0, \Delta c_o(t)=0, \Delta c_e(t)=b,~~ t=n\gamma ,~~ n\in Z^+. \end{aligned}\right. \end{aligned}$$

Because the solutions of $c_0(t)$ and $c_e(t)$ can be solved by the third and fourth equations of (3), we only consider the following system

4 $$\begin{aligned} \left\{ \begin{aligned}{}&\left. \begin{aligned}{}&dx_1(t)=x_1(t)[r_{1e}+(r_{10}-r_{1e})e^{-\mu _1 t}-\delta c_o(t)-a_1x_1(t)]dt+[d_{21}x_2(t)\\&~~~~~~~~-d_{12}(1+\frac{\rho c_e^2(t)}{1+\alpha c_e^2(t)})x_1(t)]dt+\sigma _1(t)x_1(t)dB_1(t),\\&dx_2(t)=x_2(t)[r_{2e}+(r_{20}-r_{2e})e^{-\mu _2 t}-a_2x_2(t)]dt+[d_{12}(1+\frac{\rho c_e^2(t)}{1+\alpha c_e^2(t)})x_1(t)\\&~~~~~~~~-d_{21}x_2(t)]dt+\sigma _2(t)x_2(t)dB_2(t).\\ \end{aligned}\right. \end{aligned}\right. \end{aligned}$$

### *Remark 1*

Because each of $c_o(t)$ and $c_e(t)$ is a concentration, $c_o(t)$ and $c_e(t)$ must satisfy the inequalities $0\le c_o(t)\le 1$ and $0\le c_e(t)\le 1$ for $t\ge 0$. Therefore, throughout this article, we assume that $f\le g+m$ and $b\le 1-e^{-h\gamma }$.

## Preliminaries

For the convenience of later discussion, some notations are defined here:

$$\begin{aligned} \mathbb {R}_+^2= & {} \{(x_1,x_2)|x_i>0,i=1,2.\}, \langle f(t)\rangle =t^{-1}\int _0^tf(s)ds, f^*=\limsup \limits _{t\rightarrow +\infty }f(t), f_*=\liminf \limits _{t\rightarrow +\infty }f(t),\\ \eta= & {} \frac{1}{2h\gamma \alpha }\ln \frac{(1-e^{-h\gamma })^2+\alpha b^2}{(1-e^{-h\gamma })^2+\alpha b^2e^{-2h\gamma }}, r_1(t)=r_{1e}+(r_{10}-r_{1e})e^{-\mu _1 t}-\delta c_o(t), r_1^*=r_{1e}-\delta c_o^m,\\ d_{12}(t)= & {} d_{12}\left( 1+\frac{\rho c_e^2(t)}{1+\alpha c_e^2(t)}\right) , r_2(t)=r_{2e}+(r_{20}-r_{2e})e^{-\mu _2 t}, (r_1)_*=r_{1e}-\delta c_o^M,\\ (d_{12})_*= & {} d_{12}\left( 1+\frac{\rho (c_e^m)^2}{1+\alpha (c_e^m)^2}\right) , d_{12}^*=d_{12}\left( 1+\frac{\rho (c_e^M)^2}{1+\alpha (c_e^M)^2}\right) , \sigma ^2=\frac{\xi _1^2\xi _2^2}{2\mu _1\xi _2^2+2\mu _2\xi _1^2},\hat{\sigma }^2\\= & {} \max \left\{ \frac{\xi _1^2}{2\mu _1}, \frac{\xi _2^2}{2\mu _2}\right\} , \end{aligned}$$

where $c_o^m$, $c_o^M$, $c_e^m$ and $c_e^M$ are given in Lemma 1.

### Definition 1

(*see*^31^)

(i) The population *x* is said to go to extinction if $\lim \limits _{t\rightarrow +\infty }x(t)=0$.
(ii) The population *x* is said to be strongly persistent in the mean if $\langle x(t)\rangle _*>0$.
(iii) The population *x* is said to be stochastically permanent if for any $\epsilon \in (0, 1)$, there exist $H_1=H_1(\epsilon )>0$ and $H_2=H_2(\epsilon )>0$ such that $\liminf \limits _{t\rightarrow +\infty }\mathbb {P}\{|x(t)|>H_1\}\ge 1-\epsilon ,~ \liminf \limits _{t\rightarrow +\infty }\mathbb {P}\{|x(t)|<H_2\}\ge 1-\epsilon$.

### Lemma 1

(*see*^31^) *Consider the following model corresponding to model* (3)

5 $$\begin{aligned} \left\{ \begin{aligned}{}&\frac{dc_o(t)}{dt}=fc_e(t)-(g+m)c_o(t),\\&\frac{dc_e(t)}{dt}=-hc_e(t),~~t\ne n\gamma ,\\&\Delta c_e(t)=b,~~t=n\gamma . \end{aligned}\right. \end{aligned}$$

Model (5) has a unique globally asymptotically stable positive $\gamma$-periodic solution $(\widetilde{c_o(t)}, \widetilde{c_e(t)})$, where

$$\begin{aligned} \left\{ \begin{aligned} \widetilde{c_o(t)}&=\widetilde{c_o(0)}e^{-(g+m)(t-n\gamma )}+\frac{fb(e^{-(g+m)(t-n\gamma )}-e^{-h(t-n\gamma )})}{(h-g-m)(1-e^{-h\gamma })},\\ \widetilde{c_e(t)}&=\frac{be^{-h(t-n\gamma )}}{1-e^{-h\gamma }},\\ {}\widetilde{c_o(0)}&=\frac{fb(e^{-(g+m)\gamma }-e^{-h\gamma })}{(h-g-m)(1-e^{-h\gamma })(1-e^{-(g+m)\gamma })},\\ \widetilde{c_e(0)}&=\frac{b}{1-e^{-h\gamma }}, \end{aligned}\right. \end{aligned}$$

and $$c_o^m=\inf \limits _{t\ge 0}\{\widetilde{c_o(t)}\}$$, $$c_o^M=\sup \limits _{t\ge 0}\{\widetilde{c_o(t)}\}$$, $$c_e^M=\frac{b}{1-e^{-h\gamma }}$$ and $$c_e^m=\frac{be^{-h\gamma }}{1-e^{-h\gamma }}$$.

### Lemma 2

*The positive* $\gamma$*-periodic solution* ($\widetilde{c_o(t)}$, $\widetilde{c_e(t)}$) *of model* (5) satisfies

$$\begin{aligned} \begin{aligned}{}&\lim \limits _{t\rightarrow +\infty }t^{-1}\int _0^t\widetilde{c_o(s)}ds=\frac{fb}{h(g+m)\gamma }, ~\lim \limits _{t\rightarrow +\infty }t^{-1}\int _0^t\frac{\widetilde{c_e^2(s)}}{1+\alpha \widetilde{c_e^2(s)}}ds=\eta . \end{aligned} \end{aligned}$$

### *Proof*

It follows from the periodicity of $\widetilde{c_o(t)}$ and $\widetilde{c_e(t)}$ that

$$\begin{aligned} \lim \limits _{t\rightarrow +\infty }t^{-1}\int _0^t\widetilde{c_o(s)}ds=\gamma ^{-1}\int _0^\gamma \widetilde{c_o(s)}ds=\frac{fb}{h(g+m)\gamma }, \end{aligned}$$

and

$$\begin{aligned}\begin{aligned} \lim \limits _{t\rightarrow +\infty }t^{-1}\int _0^t\frac{(\widetilde{c_e(s)})^2}{1+\alpha (\widetilde{c_e(s)})^2}ds&=\frac{-1}{\gamma h}\int _0^\gamma \frac{-h(\widetilde{c_e(s)})^2}{1+\alpha (\widetilde{c_e(s)})^2}ds=\frac{-1}{\gamma h}\int _0^\gamma \frac{\widetilde{c_e(s)}}{1+\alpha (\widetilde{c_e(s)})^2}d\widetilde{c_e(s)}\\&=\frac{1}{2h\gamma \alpha }\ln \frac{(1-e^{-h\gamma })^2+\alpha b^2}{(1-e^{-h\gamma })^2+\alpha b^2e^{-2h\gamma }}=\eta . \end{aligned} \end{aligned}$$

This result is confirmed. $\square$

## Main results

In order to study the long-time behaviors of the model (4), we first discuss the existence and uniqueness of global positive solutions to the stochastic differential equation (SDE) (4).

### Existence and uniqueness of the positive solution for SDE (4)

#### Theorem 1

For any given initial value $x(0)=(x_1(0), x_2(0))\in \mathbb {R}_+^2,$ there exists a unique global positive solution $x(t)=(x_1(t), x_2(t))$ to SDE (4), and the solution *x*(*t*) will remain $\mathbb {R}_+^2$ with probability 1.

#### *Proof*

Because the coefficients of the SDE (4) are locally *Lipschitz* continuous, there must be a unique local solution *x*(*t*) in $[0,\tau _e)$ for any given initial value $x(0)\in \mathbb {R}_+^2$, where $\tau _e$ denotes the explosion time. Therefore, we need to prove $\tau _e=+\infty ~a.s.$ in the following. Let $N_0$ be large enough such that *x*(0) remains in the interval $[\frac{1}{N_0}, N_0]$. For every $N\ge N_0$, define the stopping time

$$\begin{aligned} \tau _N=\inf \left\{ t\in [0,\tau _e]: x_i(t)\notin \left( \frac{1}{N}, N\right) , i=1,~2.\right\} . \end{aligned}$$

Clearly, $\tau _N$ is increasing as $N\rightarrow +\infty$. Letting $\tau _{\infty }=\lim \limits _{N\rightarrow \infty }\tau _N$, thus, $\tau _{\infty }\le \tau _e ~a.s.$ In the following, we only need to prove $\tau _{\infty }=+\infty ,~ a.s.$ We next employ the reduction to absurdity to prove it. If the conclusion is not true, then there are $T>0$ and $\epsilon \in (0, 1)$ such that $P\{\tau _{\infty }<T\}>\epsilon$. Accordingly, there is a positive integer $N_1\ge N_0$ such that for any $N\ge N_1$, $P\{\tau _{N}\le T\}\ge \epsilon$. Define a $C^2$-function $V: \mathbb {R}_+^2\rightarrow \mathbb {R}_+$ as follows:

$$\begin{aligned} V(x_1, x_2)=\left[ x_1-1-\ln x_1\right] +\left[ x_2-1-\ln x_2\right] . \end{aligned}$$

Using $It\hat{o}'s$ formula, we have

6 $$\begin{aligned} dV(x)=LV(x)dt+\sigma _1(t)(x_1-1)dB_1(t)+\sigma _2(t)(x_2-1)dB_2(t), \end{aligned}$$

here

$$\begin{aligned} \begin{aligned} LV(x)&=\left[ r_{1e}+(r_{10}-r_{1e})e^{-\mu _1 t}-\delta c_o+a_1\right] x_1-a_1x_1^2+\left[ r_{2e}+(r_{20}-r_{2e})e^{-\mu _2 t}+a_2\right] x_2-a_2x_2^2\\&\quad -\left[ r_{1e}+(r_{10}-r_{1e})e^{-\mu _1 t}-\delta c_o\right] -\left[ r_{2e}+(r_{20}-r_{2e})e^{-\mu _2 t}\right] +d_{12}\left( 1+\frac{\rho c_e^2}{1+\alpha c_e^2}\right) \\&\quad +d_{21}+0.5\sigma _1^2(t)+0.5\sigma _2^2(t)-d_{12}\left( 1+\frac{\rho c_e^2}{1+\alpha c_e^2}\right) \frac{x_1}{x_2}-d_{21}\frac{x_2}{x_1}\\&\le \left[ r_{1e}+r_{10}+a_1\right] x_1-a_1x_1^2+\left[ r_{2e}+r_{20}+a_2\right] x_2-a_2x_2^2+\delta c_o(t)+d_{12}(1+\rho )+d_{21}\\&\quad +\frac{\xi _1^2}{4\mu _1}+\frac{\xi _2^2}{4\mu _2}.\\ \end{aligned} \end{aligned}$$

Obviously, there exists $K>0$ such that $LV(x)\le K$.

Integrating (6) on $[0, \tau _N\wedge T]$, and then taking expectation obtain that

7 $$\begin{aligned} \mathbb {E}V(x(\tau _N\wedge T))\le V(x(0))+K T. \end{aligned}$$

Let $\Omega _N=\{\tau _N\le T\},~N>N_1$, then $P(\Omega _N)\ge \epsilon$. For any $\omega \in \Omega _N$, we get that at least one of $x_1(\tau _N, \omega )$ and $x_2(\tau _N, \omega )$ equals either *N* or $\frac{1}{N}$, thus

$$\begin{aligned} V(x(\tau _N\wedge T))\ge \min \left\{ N-1+\ln N, \frac{1}{N}-1-\ln N\right\} . \end{aligned}$$

From (7), we have

$$\begin{aligned} V(x(0))+KT\ge \mathbb {P}(\Omega _N)V(x(\tau _N\wedge T))\ge \epsilon \min \left\{ N-1+\ln N, \frac{1}{N}-1-\ln N\right\} . \end{aligned}$$

Letting $N\rightarrow +\infty$, leads to the contradiction:

$$\begin{aligned} +\infty >V(x(0))+KT\ge \mathbb {P}(\Omega _N)V(x(\tau _N\wedge T))\ge \epsilon \min \{N-1+\ln N, \frac{1}{N}-1-\ln N\}=\infty . \end{aligned}$$

Therefore, we obtain $\tau _{\infty }=+\infty$, a.s. $\square$

### Stochastic permanence

#### Lemma 3

For any given initial value $x(0)\in \mathbb {R}_+^2$, there must be a $K(p)>0$ such that the solution *x*(*t*) of SDE (4) satisfies

$$\begin{aligned} \limsup \limits _{t\rightarrow +\infty } \mathbb {E}[(x_1+x_2)^p]\le K(p),~ p>1. \end{aligned}$$

#### Proof

Define function $V(x)=(x_1+x_2)^p$, $(p>1)$, using It$\hat{o}$’s formula to *V*(*x*), we obtain

$$\begin{aligned} \begin{aligned} dV(x)&=p(x_1+x_2)^{p-1}d(x_1+x_2)+\frac{1}{2}p(p-1)(x_1+x_2)^{p-2}(d(x_1+x_2))^2\\&\le p(x_1+x_2)^{p-1}\{x_1[r_{1e}+r_{10}-a_1x_1]+x_2[r_{2e}+r_{20}-a_2x_2]\}dt\\&\quad +\frac{1}{2}p(p-1)(x_1+x_2)^{p-2}\left[ x_1^2\frac{\xi _1^2}{2\theta _1}+x_2^2\frac{\xi _2^2}{2\theta _2}\right] dt+p(x_1+x_2)^{p-1}(\sigma _1(t)x_1dB_1(t)+\sigma _2(t)x_2dB_2(t))\\&\le (x_1+x_2)^{p}\{r-a(x_1+x_2)\}dt+p(x_1+x_2)^{p-1}(\sigma _1(t)x_1dB_1(t)+\sigma _2(t)x_2dB_2(t)),\\ \end{aligned} \end{aligned}$$

where $$r=p\max \{r_{1e}+r_{10}, r_{2e}+r_{20}\}+\frac{1}{2}p(p-1)\max \left\{ \frac{\xi _1^2}{2\theta _1},\frac{\xi _2^2}{2\theta _2}\right\}$$, $$a=\frac{p\min \{a_1,a_2\}}{2}$$. Thus,

$$\begin{aligned} \mathbb {E}V(x(t))-V(x(0))\le \int _{0}^{t}r\mathbb {E}V(x(s))-a\mathbb {E}V^{\frac{p+1}{p}}(x(s))ds, \end{aligned}$$

further,

8 $$\begin{aligned} \frac{d\mathbb {E}V(x(t))}{dt}\le \mathbb {E}V(x(t))\left( r-a(\mathbb {E}V(x(t)))^{\frac{1}{p}}\right) . \end{aligned}$$

Let $y(t)=\mathbb {E}V(x(t))$, from (8), we have

$$\begin{aligned} \frac{dy(t)}{dt}\le y(t)\left( r-ay^{\frac{1}{p}}(t)\right) . \end{aligned}$$

By the comparison theorem, we obtain $\limsup \limits _{t\rightarrow +\infty }y(t)\le (\frac{r}{a})^p$, that is, $\limsup \limits _{t\rightarrow +\infty }\mathbb {E}(x_1(t)+x_2(t))^p\le (\frac{r}{a})^p=K(p)$. This ends the proof. $\square$

#### *Remark 2*

From Lemma 3, we know that there exists a $T>0$ such that $\mathbb {E}[(x_1(t)+x_2(t))^p]\le 2K(p)$ for $t>T$. On the other hand, $\mathbb {E}[(x_1(t)+x_2(t))^p]$ is continuous with respect to *t* on the interval [0, *T*], then there exists a $K_1(p)>0$ such that $\mathbb {E}[(x_1(t)+x_2(t))^p]\le K_1(p)$ for $t\in [0, T]$. Let $K_0(p)=\max \{2K(p), K_1(p)\},$ we have $\mathbb {E}[(x_1(t)+x_2(t))^p]\le K_0(p)$, that is, the solution *x*(*t*) to SDE (4) is P-moment bounded.

#### Theorem 2

If $\min \{r_{1e}-\delta c_M, r_{2e}\}>0.5\hat{\sigma }^2$, the solution *x*(*t*) of SDE (4) is stochastically permanent.

#### Proof

Define function $V_1(x)=x_1(t)+x_2(t)$, $t\ge 0$, we can obtain that

$$\begin{aligned} dV_1(x)=\left[ x_1(t)(r_1(t)-a_1x_1(t))+x_2(t)(r_2(t)-a_2x_2(t))\right] dt+\sigma _1(t)x_1(t)dB_1(t)+\sigma _2(t)x_2(t)dB_2(t). \end{aligned}$$

Define function $U(x)=\frac{1}{V_1(x)}$, $t\ge 0$. Applying It$\hat{o}$’s formula, we have

9 $$\begin{aligned} \begin{aligned} dU&=-U^2dV_1+U^3(dV_1)^2\\&=-U^2[x_1(t)(r_1(t)-a_1x_1(t))+x_2(t)(r_2(t)-a_2x_2(t))]dt\\&\quad +U^3(\sigma ^2_1(t)x_1^2+\sigma ^2_2(t)x_2^2)dt-U^2(\sigma _1(t)x_1(t)dB_1(t)+\sigma _2(t)x_2(t)dB_2(t)). \end{aligned} \end{aligned}$$

If $\min \{r_{1e}-\delta c_M, r_{2e}\}>0.5\hat{\sigma }^2$, we can take an $\epsilon >0$ small enough such that $\check{r}=\min \{(r_1(t))_*,(r_2(t))_*\}=\min \{r_{1e}-\delta c_M, r_{2e}\}>0.5\hat{\sigma }^2+\epsilon$. Moreover, we can also select a $\theta >0$ such that $(\check{r}-\epsilon )-0.5(\theta +1)\hat{\sigma }^2>0$. Define function $V_2(t)=(1+U(x))^{\theta }$. An application of It$\hat{o}$’s formula gives

$$\begin{aligned} \begin{aligned} dV_2(x)&=\theta (1+U(x))^{\theta -1}dU(x)+\frac{1}{2}\theta (\theta -1)(1+U(x))^{\theta -2}(dU(x))^2\\&=LV_2(x)dt-\theta (1+U)^{\theta -1}U^2(\sigma _1(t)x_1(t)dB_1(t)+\sigma _2(t)x_2(t)dB_2(t)), \end{aligned} \end{aligned}$$

here $$\hat{a}=\max \{a_1,a_2\}$$ and

10 $$\begin{aligned} \begin{aligned} LV_2(x)&=\theta (1+U)^{\theta -1}\{-U^2\{x_1[r_1(t)-a_1x_1]+x_2[r_2(t)-a_2x_2]\}+U^3(\sigma ^2_1(t)x_1^2+\sigma ^2_2(t)x_2^2)\}\\&\quad +\frac{1}{2}\theta (\theta -1)(1+U)^{\theta -2}U^4(\sigma _1^2(t)x_1^2+\sigma _2^2(t)x_2^2)\\&\le (1+U)^{\theta -2}\{\theta U^3\{-x_1(r_{1e}-\epsilon -\delta c_M)-x_2(r_{2e}-\epsilon )+\theta (1+U)U^2(a_1x_1^2+a_2x_2^2)\}\\&\quad +(\theta (1+U)U^3+\frac{1}{2}\theta (\theta -1)U^4)\hat{\sigma }(x_1^2+x_2^2)\}\\&\le (1+U)^{\theta -2}\left\{ \left[ \frac{1}{2}\theta (\theta +1)\hat{\sigma }-(\check{r}-\epsilon )\theta \right] U^2+(\theta \hat{a}+\theta \hat{\sigma })U+\theta \hat{a}\right\} \end{aligned} \end{aligned}$$

for *t* large enough. We select a $\zeta >0$ small enough to satisfy

11 $$\begin{aligned} (\check{r}-\epsilon )\theta -\frac{1}{2}\theta (\theta +1)\hat{\sigma }-\zeta >0, \end{aligned}$$

By computing, we have

12 $$\begin{aligned} \mathbb {E}[e^{\zeta t}V_2(x)]=V_2(x(0))+\mathbb {E}\int _{0}^{t}L[e^{\zeta s}V_2(x(s))]ds, \end{aligned}$$

where

13 $$\begin{aligned} \begin{aligned} L[e^{\zeta t}V_2(x)]&=\eta e^{\zeta t}V_2(x)+e^{\zeta t}LV_2(x)\\&\le e^{\zeta t}(1+U)^{\theta -2}\left\{ \zeta (1+U)^2+\left[ \frac{1}{2}\theta (\theta +1)\hat{\sigma }-(\check{r}-\epsilon )\theta \right] U^2+(\theta \hat{a}+\theta \hat{\sigma })U+\theta \hat{a}\right\} \\&=e^{\zeta t}(1+U)^{\theta -2}\left\{ -\left[ (\check{r}-\epsilon )\theta -\frac{1}{2}\theta (\theta +1)\hat{\sigma }-\zeta \right] U^2+(\theta \hat{a}+\theta \hat{\sigma }+2\zeta )U+\theta \hat{a}+\zeta \right\} \\&\le \zeta e^{\zeta t}\kappa (x), \end{aligned} \end{aligned}$$

here

$$\begin{aligned} \kappa (x)=\frac{1}{\zeta }\{(1+U)^{\theta -2}\{-[(\check{r}-\epsilon )\theta -\frac{1}{2}\theta (\theta +1)\hat{\sigma }-\zeta ]U^2+(\theta \hat{a}+\theta \hat{\sigma }+2\zeta )U+\theta \hat{a}+\zeta \}\}. \end{aligned}$$

From (11), we know that $\kappa (x)$ is bounded in $\mathbb {R}_+^2$. Let $\kappa _1=\max \left\{ \sup \limits _{x\in R_+^2} \kappa (x), 1\right\} <+\infty .$ It follows from (12) that

$$\begin{aligned} \mathbb {E}[e^{\zeta t}V_2(x)]\le V_2(x(0))+\kappa _1(e^{\zeta t}-1) \end{aligned}$$

for *t* large enough. Further, we can obtain that

$$\begin{aligned} \limsup \limits _{t\rightarrow +\infty } \mathbb {E}\frac{1}{(x_1+x_2)^\theta }\le \limsup \limits _{t\rightarrow +\infty } \mathbb {E}\left( 1+\frac{1}{(x_1+x_2)^\theta }\right) \le \kappa _1. \end{aligned}$$

For any $\epsilon \in (0, 1)$, denote $H_1=\epsilon ^{\theta }/\kappa _1^\theta$. By Chebyshev’s inequality (see^23^), we can obtain that

$$\begin{aligned} \mathbb {P}\{(x_1(t)+x_2(t))<H_1\}=\mathbb {P}\left\{ \frac{1}{(x_1(t)+x_2(t))^\theta }>\frac{1}{H_1^\theta }\right\} \le H_1^\theta \mathbb {E}\frac{1}{(x_1(t)+x_2(t))^\theta }, \end{aligned}$$

thus $$\limsup \limits _{t\rightarrow +\infty }\mathbb {P}\{(x_1(t)+x_2(t))<H_1\}\le \epsilon ,$$ and $$\liminf \limits _{t\rightarrow +\infty }\mathbb {P}\{(x_1(t)+x_2(t))>H_1\}\ge 1-\epsilon .$$

We will prove in the following that for any $\epsilon >0$, there is a $H_2(\epsilon )>0$ such that $\liminf \limits _{t\rightarrow +\infty }\mathbb {P}\{(x_1(t)+x_2(t))\le H_2\}\ge 1-\epsilon .$ According to Lemma 3 and the Chebyshev’s inequality, this result can be easily confirmed. $\square$

### Extinction

#### Lemma 4

The solution *x*(*t*) to SDE (4) satisfies $\limsup \limits _{t\rightarrow +\infty }\frac{\ln x_i(t)}{t}\le 0,~~a.s.,~i=1,2.$

#### *Proof*

Define function $V_3(x)=\ln (x_1+\theta x_2)$  $(\theta >0)$. Applying $It\hat{o}$’s formula for $V_3(x)$, we have

14 $$\begin{aligned} \begin{aligned} d\ln (x_1+\theta x_2)&=\left( \frac{x_1[r_1(t)-a_1x_1]+[d_{21}x_2-d_{12}(t)x_1]}{x_1+\theta x_2}+\frac{\theta x_2[r_2(t)-a_2x_2]+\theta [d_{12}(t)x_1-d_{21}x_2]}{x_1+\theta x_2}\right. \\&\quad -\left. \frac{\sigma _1^2(t)x_1^2+\sigma _2^2(t)\theta ^2 x_2^2}{2(x_1+\theta x_2)^2}\right) dt +\frac{\sigma _1(t)x_1dB_1(t)+\sigma _2(t)\theta x_2dB_2(t)}{x_1+\theta x_2}\\&\le \left( \frac{(r_{1e}+r_{10}+\theta d_{12}(1+\rho ))x_1+(r_{2e}+r_{20}+\frac{d_{21}}{\theta })\theta x_2-a_1x_1^2-\theta a_2x_2^2}{x_1+\theta x_2}\right. \\&\quad \left. -\frac{\sigma _1^2(t)x_1^2+\sigma _2^2(t)\theta ^2 x_2^2}{2(x_1+\theta x_2)^2}\right) dt+\frac{\sigma _1(t)x_1dB_1(t)+\sigma _2(t)\theta x_2dB_2(t)}{x_1+\theta x_2}\\&\le \left( r-\nu (x_1+\theta x_2) -\frac{\sigma _1^2(t)x_1^2+\sigma _2^2(t)\theta ^2x_2^2}{2(x_1+\theta x_2)^2}\right) dt+\frac{\sigma _1(t)x_1dB_1(t)+\sigma _2(t)\theta x_2dB_2(t)}{x_1+\theta x_2},\\ \end{aligned} \end{aligned}$$

where $r=\max \{r_{1e}+r_{10}+\theta d_{12}(1+\rho ), (r_{2e}+r_{20}+\frac{d_{21}}{\theta })\}$ and ${\nu }=0.5\min \{a_1, \frac{a_2}{\theta }\}$. Thus,

15 $$\begin{aligned} \begin{aligned} de^tV(x)&=e^tV(x)dt+e^tdV(x)\\&\le e^t\left( \ln (x_1+\theta x_2)+r-\nu (x_1+\theta x_2)-\frac{\sigma _1^2(t)x_1^2+\sigma _2^2(t)\theta ^2 x_2^2}{2(x_1+\theta x_2)^2}\right) dt\\&\quad +e^t\frac{\sigma _1(t)x_1dB_1(t)+\sigma _2(t)\theta x_2dB_2(t)}{x_1+\theta x_2}. \end{aligned} \end{aligned}$$

Integrating both sides of inequality (15) in the interval [0, *t*], we have

16 $$\begin{aligned} \begin{aligned} e^tV(x)&\le V(x(0))+\int _0^te^s(\ln (x_1(s)+\theta x_2(s))+{r}-\nu (x_1(s)+\theta x_2(s))\\&\quad -\frac{\sigma _1^2(t)x_1^2(s)+\sigma _2^2(t)\theta x_2^2(s)}{2(x_1(s)+\theta ^2 x_2(s))^2})ds+M(t), \end{aligned} \end{aligned}$$

where $M(t)=\int _0^t e^s\frac{\sigma _1(t)x_1(s)dB_1(s)+\sigma _2(t)\theta x_2(s)dB_2(s)}{x_1(s)+\theta x_2(s)}$. The quadratic variation of *M*(*t*) is $\langle M(t), M(t)\rangle _t=\int _0^te^{2s}\frac{\sigma _1^2(t)x_1^2(s)+\sigma _2^2(t)\theta ^2x_2^2(s)}{(x_1(s)+\theta x_2(s))^2}ds$. According to the exponential martingale inequality, for all positive constants $\varepsilon , \beta$ and $T_0$, we can obtain that

$$\begin{aligned} \mathbb {P}\left\{ \sup \limits _{0\le t\le T_0}[M(t)-0.5\varepsilon \langle M(t), M(t)\rangle _t]>\beta \right\} \le e^{-\varepsilon \beta }, \end{aligned}$$

and choose $\varepsilon =e^{-n}$, $\beta =2e^n\ln n$ and $T_0=n$, then

$$\begin{aligned} \mathbb {P}\left\{ \sup \limits _{0\le t\le n}[M(t)-0.5e^{-n}\langle M(t), M(t)\rangle _t]>2 e^n\ln n\right\} \le n^{-2}. \end{aligned}$$

Because the series $\sum \limits _{n=1}^{+\infty } n^{-2}<\infty$, by Borel-Cantalli lemma, we obtain that there is a $\Omega _0\in \Omega$ with $\mathbb {P}(\Omega _0)=1$ such that for every $\omega \in \Omega _0$, a positive integer $n_1=n_1(\omega )$ can be found that

17 $$\begin{aligned} M(t)\le 0.5e^{-n}\langle M(t), M(t)\rangle _t+2e^n\ln n \end{aligned}$$

for $0\le t\le n$ and $n\ge n_1(\omega )$. Let $\varphi (x)=\ln (x_1+\theta x_2)+{r}-\nu (x_1+\theta x_2)$, we obtain that there must be a positive constant *K* such that $\varphi (x)\le K$ for $x\in \mathbb {R}_+^2$. It follows from (16) and (17) that for all $n>n_1(\omega )$,

18 $$\begin{aligned} e^t\ln (x_1+\theta x_2)\le V(x(0))+K(e^t-1)+2 e^n\ln n. \end{aligned}$$

If $n-1\le t\le n$ and $n>n_1(\omega )$, we have

$$\begin{aligned} \frac{\ln (x_1+\theta x_2)}{t}\le \frac{V(x(0))}{te^t}+\frac{K(e^t-1)}{t e^t}+\frac{2 e^n\ln n}{te^t}. \end{aligned}$$

Letting $t\rightarrow +\infty$, we can obtain that $\limsup \limits _{t\rightarrow +\infty }\ln \frac{\ln (x_1(t)+\theta x_2(t))}{t}\le 0,~a.s.$, this can also imply that $\limsup \limits _{t\rightarrow +\infty }\ln \frac{\ln x_i(t)}{t}\le 0,~a.s.,~i=1,2.$ when we take $\theta =1$.

This completes the proof. $\square$

#### Theorem 3

Let *x*(*t*) be a solution of SDE (4) with initial value $x(0)\in R_+^2$. If any of the following conditions is true,

(i): $$r_1^*=r_{2e}$$ and $$r_1^*+r_{2e}<\sigma ^2$$.
(ii): $$r_1^*<r_{2e}$$ and $$(r_1^*+r_{2e}-d^*_{12}-d_{21})+\sqrt{(r_1^*-r_{2e}+d_{21}-d^*_{12})^2+4d^*_{12}d_{21}}<\sigma ^2$$.
(iii): $r_1^*>r_{2e}$ and $(r_1^*+r_{2e}-d_{21}-(d_{12})_*)+\sqrt{(r_1^*-r_{2e}+d_{21}-(d_{12})_*)^2+4(d_{12})_*d_{21}}<\sigma ^2$. Then the single-species population goes to die out, that is, $\lim \limits _{t\rightarrow +\infty }x_i(t)=0,~~a.s.$

#### Proof

From SDE (4), we obtain that

19 $$\begin{aligned} \begin{aligned} d(x_1+\theta x_2)&=\left[ (r_1(t)+d_{12}(t)(\theta -1))x_1 +(\theta r_2(t)+ d_{21}(1-\theta ))x_2 -a_1x_1^2-a_2\theta x_2^2\right] dt\\&\quad +\sigma _1(t)x_1dB_1(t)+\theta \sigma _2(t)x_2dB_2(t). \end{aligned} \end{aligned}$$

From Lemma 1, we derive that for $\epsilon >0$, there exists a $T_1>0$ such that for $t\ge T_1$,

20 $$\begin{aligned} \begin{aligned} (r_1)_*-\epsilon&=r_{1e}-\delta c_o^M-\epsilon \le r_1(t)\le r_{1e}-\delta c_m+\epsilon =r_1^*+\epsilon ,~ r_{2e}-\epsilon \le r_2(t)\le r_{2e}+\epsilon ,\\ (d_{12})_*-\epsilon&=d_{12}\left( 1+\frac{\rho _1 (c_e^m)^2}{1+\alpha (c_e^m)^2}\right) -\epsilon \le d_{12}(t)\le d_{12}\left( 1+\frac{\rho _1 (c_e^M)^2}{1+\alpha (c_e^M)^2}\right) +\epsilon =d_{12}^*+\epsilon . \end{aligned} \end{aligned}$$

*Case* (*i*) :  If $r_1^*=r_{2e}$, we take $\theta =1$, and obtain from (19) that

21 $$\begin{aligned} \begin{aligned} d(x_1+x_2)&=\left[ (r_1(t)x_1+r_2(t) x_2)-a_1 x_1^2-a_2 x_2^2\right] dt+\sigma _1(t)x_1dB_1(t)+\sigma _2(t)x_2dB_2(t)\\&\le (r_{1}^*+\epsilon )(x_1+x_2)dt+\sigma _1(t)x_1dB_1(t)+\sigma _2(t)x_2dB_2(t). \end{aligned} \end{aligned}$$

Applying $It\hat{o}'s$ formula, we have

22 $$\begin{aligned} \begin{aligned} d\ln (x_1+x_2)&\le \left( (r_{1}^*+\epsilon )-0.5\frac{\sigma _1^2(t)x_1^2+\sigma _2^2(t)x_2^2}{(x_1+x_2)^2}\right) dt+\frac{\sigma _1(t)x_1dB_1(t)+\sigma _2(t)x_2dB_2(t)}{x_1+x_2},~t\ge T_1. \end{aligned} \end{aligned}$$

By Cauchy inequality, we can obtain that

$$\begin{aligned} \left( \sigma _1^2(t)\frac{x_1^2}{(x_1+x_2)^2}+\sigma _2^2(t)\frac{x_2^2}{(x_1+x_2)^2}\right) \left( \frac{1}{\sigma _1^2(t)}+\frac{1}{\sigma _2^2(t)}\right) \ge 1. \end{aligned}$$

Further from (22), we have

$$\begin{aligned} \begin{aligned} d\ln (x_1+x_2)&\le \left( (r_{1}^*+\epsilon )-\frac{0.5\sigma _1^2(t)\sigma _2^2(t)}{\sigma _1^2(t)+\sigma _2^2(t)}\right) dt+\frac{\sigma _1(t)x_1dB_1(t)+\sigma _2(t)x_2dB_2(t)}{x_1+x_2},~t\ge T_1. \end{aligned} \end{aligned}$$

Integrating both sides of above inequality on $[T_1,t]$ and dividing by *t*, we can obtain that

23 $$\begin{aligned} \frac{\ln (x_1(t)+ x_2(t))}{t}\le \frac{\ln (x_1(T_1)+ x_2(T_1))}{t}+\frac{(r_{1}^*+\epsilon )(t-T_1)}{t}-\frac{1}{2t}\int _{T_1}^t\frac{\sigma _1^2(s)\sigma _2^2(s)}{\sigma _1^2(s)+\sigma _2^2(s)}ds+\frac{M_1(t)}{t}, \end{aligned}$$

where $M_1(t)=\int _{T_1}^t\frac{\sigma _1(s)x_1(s)dB_1(s)+\sigma _2(s)x_2(s)dB_2(s)}{x_1(s)+x_2(s)}.$ Let $N(t)=\int _0^{T_1}\frac{\sigma _1(s)x_1(s)dB_1(s)+\sigma _2(s)x_2(s)dB_2(s)}{x_1(s)+x_2(s)}+M_1(t)$, then the quadratic variation of *N*(*t*) is

$$\begin{aligned} \langle N(t),N(t)\rangle _t=\int _0^t\frac{\sigma _1^2(t)x_1^2(s)+\sigma _2^2(s)x_2^2(s)}{(x_1(s)+x_2(s))^2}ds\le \max \{\frac{\xi _1^2}{2\theta _1},\frac{\xi _2^2}{2\theta _2}\}t. \end{aligned}$$

According to the strong law of large number, we have $\lim \limits _{t\rightarrow +\infty }\frac{N(t)}{t}=0$, thus, $\lim \limits _{t\rightarrow +\infty }\frac{M(t)}{t}=0$. And

$$\begin{aligned} \begin{aligned} \int _{T_1}^t\frac{\frac{\xi _1^2\xi _2^2}{4\theta _1\theta _2}(1-e^{-2\theta _1s})(1-e^{-2\theta _2s})}{\frac{\xi _1^2}{2\theta _1}+\frac{\xi _2^2}{2\theta _2}}ds\le \int _{T_1}^t\frac{\sigma _1^2(s)\sigma _2^2(s)}{\sigma _1^2(s)+\sigma _2^2(s)}ds\le \int _{T_1}^t\frac{\frac{\xi _1^2\xi _2^2}{4\theta _1\theta _2}}{\frac{\xi _1^2}{2\theta _1}(1-e^{-2\theta _1s})+\frac{\xi _2^2}{2\theta _2}(1-e^{-2\theta _2s})}ds, \end{aligned} \end{aligned}$$

thus, $$\lim \limits _{t\rightarrow +\infty }t^{-1}\int _{T_1}^t\frac{\sigma _1^2(s)\sigma _2^2(s)}{\sigma _1^2(s)+\sigma _2^2(s)}ds=\frac{\xi _1^2\xi _2^2}{2\theta _1\xi _2^2+2\theta _2\xi _1^2}=\sigma ^2$$.

If $r_1^*+r_{2e}-\sigma ^2<0$, we can take a sufficiently small $\epsilon \in (0,1)$ such that $r_1^*+r_{2e}+2\epsilon -\sigma ^2<0$, from (23), we have $\lim \limits _{t\rightarrow +\infty }(x_1(t)+x_2(t))=0,~ a.s$, that is, $\lim \limits _{t\rightarrow +\infty }x_i(t)=0,$ $a.s.,~i=1,~2.$

*Case*(*ii*) :  If $r_1^*<r_{2e}$, we take a $\theta _1>1$, from (19), we have

24 $$\begin{aligned} \begin{aligned} d(x_1+\theta _1 x_2)&\le [((r_1^*+\epsilon )+(d_{12}^*+\epsilon )(\theta _1-1))x_1 +(\theta _1 (r_{2e}+\epsilon )+ d_{21}(1-\theta _1))x_2\\&\quad -a_1x_1^2-a_2\theta _1 x_2^2]dt +\sigma _1(t)x_1dB_1(t)+\theta _1\sigma _2(t)x_2dB_2(t). \end{aligned} \end{aligned}$$

Let $(\theta _1, \lambda _1)$ be the solution of the following equations

$$\begin{aligned} \left\{ \begin{aligned}{}&(r_1^*+\epsilon )+(\theta _1-1) (d_{12}^*+\epsilon )=\lambda _1,\\&\theta _1 (r_{2e}+\epsilon )+(1-\theta _1)d_{21}=\theta _1\lambda _1, \end{aligned}\right. \end{aligned}$$

and

25 $$\begin{aligned} \left\{ \begin{aligned} \theta _1-1&=\frac{\lambda _1-(r_1^*+\epsilon )}{d_{12}^*+\epsilon }>0,\\ 1-\theta _1&=\theta _1\frac{\lambda _1-(r_{2e}+\epsilon )}{d_{21}}<0, \end{aligned}\right. \end{aligned}$$

which implies that $r_1^*+\epsilon<\lambda _1<r_{2e}+\epsilon$. Denote $p=\lambda _1-(r_1^*+\epsilon )>0$, $q=r_1^*-r_{2e}<0$. From (25), we have

26 $$\begin{aligned} f(p)=p^2+(q+d_{12}^*+d_{21}+\epsilon )p+(d^*_{12}+\epsilon )q=0, \end{aligned}$$

it is easy to calculate that the quadratic equation (26) has two real roots:

$$\begin{aligned} p_1=\frac{-(q+d^*_{12}+d_{21}+\epsilon )+\sqrt{(q+d_{21}-d^*_{12}-\epsilon )^2+4d_{21}(d^*_{12}+\epsilon )}}{2}>0,\\p_2=\frac{-(q+d^*_{12}+d_{21}+\epsilon )-\sqrt{(q+d_{21}-d^*_{12}-\epsilon )^2+4d_{21}(d^*_{12}+\epsilon )}}{2}<0. \end{aligned}$$

And because $f(-q)=-d_{21}q>0$, it is easy to see that $0<p_1<-q$, further,

$$\begin{aligned} \left\{ \begin{aligned} \theta _1&=\frac{p_1}{d_{12}^*+\epsilon }+1>1,\\ \lambda _1&=p_1+(r_1^*+\epsilon )<r_{2e}+\epsilon . \end{aligned}\right. \end{aligned}$$

From (24), we obtain that

27 $$\begin{aligned} \begin{aligned} d(x_1+\theta _1 x_2)&\le \lambda _1(x_1+\theta _1 x_2)dt +\sigma _1(t)x_1dB_1(t)+\theta _1\sigma _2(t)x_2dB_2(t). \end{aligned} \end{aligned}$$

If $(r_1^*+r_{2e}-d^*_{12}-d_{21})+\sqrt{(r_1^*-r_{2e}+d_{21}-d^*_{12})^2+4d^*_{12}(r_{2e}-r_1^*)}<\sigma ^2$, we choose an $\epsilon$ small enough such that $\lambda _1<0.5\sigma ^2$, from (27), we also conclude that $\lim \limits _{t\rightarrow +\infty }x_i(t)=0,~a.s.,~i=1,~2.$

*Case* (*iii*): If $r_1^*>r_{2e}$, we select a $0<\theta _2<1$, from (19), we have

28 $$\begin{aligned} \begin{aligned} d(x_1+\theta _2 x_2)&\le [((r_1^*+\epsilon )+((d_{12})_*-\epsilon )(\theta _2-1))x_1 +(\theta _2 (r_{2e}+\epsilon )+ d_{21}(1-\theta _2))x_2\\&\quad -a_1x_1^2-a_2\theta _2 x_2^2]dt +\sigma _1(t)x_1dB_1(t)+\theta _2\sigma _2(t)x_2dB_2(t). \end{aligned} \end{aligned}$$

Let $(\theta _2, \lambda _2)$ be the solution of the following equations

$$\begin{aligned} \left\{ \begin{aligned}{}&(r_1^*+\epsilon )+(\theta _2-1) ((d_{12})_*-\epsilon )=\lambda _2,\\&\theta _2 (r_{2e}+\epsilon )+(1-\theta _2)d_{21}=\theta _2\lambda _2, \end{aligned}\right. \end{aligned}$$

and

29 $$\begin{aligned} \left\{ \begin{aligned} \theta _2-1&=\frac{\lambda _2-(r_1^*+\epsilon )}{(d_{12})_*-\epsilon }<0,\\ 1-\theta _2&=\theta _2\frac{\lambda _2-(r_{2e}+\epsilon )}{d_{21}}>0, \end{aligned}\right. \end{aligned}$$

this implies that $r_{2e}+\epsilon<\lambda _2<r_1^*+\epsilon$. It follows from (29) that

30 $$\begin{aligned} g(p)=p^2+(q+(d_{12})_*+d_{21}-\epsilon )p+((d_{12})_*-\epsilon )q=0, \end{aligned}$$

where $p=\lambda _2-(r_1^*+\epsilon )<0$, $q=r_1^*-r_{2e}>0$. There exist two real roots to quadratic equation $g(p)=0$,

$$\begin{aligned} p_3=\frac{-(q+d_{21}+(d_{12})_*-\epsilon )+\sqrt{(q+d_{21}-(d_{12})_*+\epsilon )^2+4((d_{12})_*-\epsilon )d_{21}}}{2}<0,\\p_4=\frac{-(q+d_{21}+(d_{12})_*-\epsilon )-\sqrt{(q+d_{21}-(d_{12})_*+\epsilon )^2+4((d_{12})_*-\epsilon )d_{21}}}{2}<0, \end{aligned}$$

moreover, $$p_4<-q<p_3<0$$, thus,

$$\begin{aligned} \left\{ \begin{aligned} \theta _2&=\frac{p_3}{(d_{12})_*-\epsilon }+1<1,\\ \lambda _2&=p_3+(r_1^*+\epsilon )>r_{2e}+\epsilon . \end{aligned}\right. \end{aligned}$$

Similar to the proof of *Case* (*ii*), we have $\lim \limits _{t\rightarrow +\infty }x_i(t)=0,~ a.s.~ (i=1,~2)$ if

$$\begin{aligned} (r_1^*+r_{2e}-d_{21}-(d_{12})_*)+\sqrt{(r_1^*-r_{2e}+d_{21}-(d_{12})_*)^2+4(d_{12})_*d_{21}}<\sigma ^2. \end{aligned}$$

This proof is completed. $\square$

#### *Remark 3*

From the proof of Theorem 3’(ii), we know that species goes to extinction when $r_1^*<0$ and $d_{12}^*r_{2e}+r_1^*d_{21}-r_1^*r_{2e}<0$, which is independent of the intensity of the noise.

#### *Remark 4*

If $\rho =0$, that is, without considering the influence of environmental toxicant concentration on population migration. From Theorem 3, we obtain that single-species population will be extinct if $(r_1^*+r_{2e}-d_{21}-d_{12})+\sqrt{(r_1^*-r_{2e}+d_{21}-d_{12})^2+4d_{12}d_{21}}<\sigma ^2$.

### Permanence in the mean

In this subsection, we aim to analyze the permanence in the mean of SDE (4).

#### Theorem 4

*Let* $(x_1(t),x_2(t))$ *be the solution of SDE* (4) *with initial value* $x(0)\in \mathbb {R}_+^2$*. If any of the following conditions is true*,

(i): $$(r_1)_*=r_{2e}$$ and $$(r_1)_*+r_{2e}>\hat{\sigma }^2$$.
(ii): $$(r_1)_*<r_{2e}$$ and $$(r_1)_*+r_{2e}-(d_{12})_*-d_{21}+\sqrt{((r_1)_*-r_{2e}+d_{21}-(d_{12})_*)^2+4(d_{12})_*d_{21}}>\hat{\sigma }^2$$.
(iii): $(r_1)_*>r_{2e}$ and $(r_1)_*+r_{2e}-d^*_{12}-d_{21}+\sqrt{((r_1)_*-r_{2e}+d_{21}-d^*_{12})^2+4d_{12}^*d_{21}}>\hat{\sigma }^2$. Then the single-species population is strongly persistent in the mean.

#### *Proof*

Using the same proof method as Theorem 3. Let $\epsilon \in (0, 1)$ be small enough, and $r_{2e}-\epsilon >0$, $(d_{21})_*-\epsilon >0$.

*Case* (*i*) :  If $(r_1)_*=r_{2e}$, we take $\theta =1$, and obtain from (19) that

31 $$\begin{aligned} \begin{aligned} d(x_1+x_2)&=\left[ (r_1(t)x_1+r_2(t) x_2)-a_1x_1^2-a_2 x_2^2\right] dt+\sigma _1(t)x_1dB_1(t)+\sigma _2(t)x_2dB_2(t)\\&\ge ((r_{2e}-\epsilon )(x_1+x_2)-a(x_1+x_2)^2)dt+\sigma _1(t)x_1dB_1(t)+\sigma _2(t)x_2dB_2(t). \end{aligned} \end{aligned}$$

Applying $It\hat{o}'s$ formula, we have

$$\begin{aligned} \begin{aligned} d\ln (x_1+x_2)&\ge ((r_{2e}-\epsilon )-0.5\hat{\sigma }^2-a(x_1+x_2))dt+\frac{\sigma _1(t)x_1dB_1(t)+\sigma _2(t)x_2dB_2(t)}{x_1+x_2},~~~t\ge T_1, \end{aligned} \end{aligned}$$

where $\hat{\sigma }^2=\max \{\frac{\xi _1}{2\theta _1}, \frac{\xi _2}{2\theta _2}\}$ and $a=\frac{\max \{a_1,a_2\}}{2}$. And,

32 $$\begin{aligned} a\langle x_1+x_2\rangle \ge -\frac{\ln (x_1(t)+x_2(t))}{t}+\frac{\ln (x_1(T_1)+x_2(T_1))}{t}+\frac{((r_{2e}-\epsilon )-0.5\hat{\sigma }^2)(t-T_1)}{t}+\frac{M(t)}{t}. \end{aligned}$$

If $(r_1)_*+r_{2e}>\hat{\sigma }$, we select a sufficiently small $\epsilon$ such that $(r_1)_*+r_{2e}-2\epsilon >\hat{\sigma }$, from Lemma 4 and (32), we obtain that

$$\begin{aligned} \langle x_1(t)+x_2(t)\rangle _*\ge \frac{(r_{2e}-\epsilon )-0.5\hat{\sigma }^2}{a}>0. \end{aligned}$$

*Case*(*ii*) :  If $(r_1)_*<r_{2e}$, we take a $\theta _3>1$, from (19), we have

33 $$\begin{aligned} \begin{aligned} d(x_1+\theta _3 x_2)&\ge [(((r_1)_*-\epsilon )+((d_{12})_*-\epsilon )(\theta _3-1))x_1 +(\theta _3 (r_{2e}-\epsilon )+ d_{21}(1-\theta _3))x_2\\&\quad -a_1x_1^2-a_2\theta _3 x_2^2]dt +\sigma _1(t)x_1dB_1(t)+\theta _3\sigma _2(t)x_2dB_2(t). \end{aligned} \end{aligned}$$

Let $(\theta _3, \lambda _4)$ be the solution of the following equations

$$\begin{aligned} \left\{ \begin{aligned}{}&((r_1)_*-\epsilon )+(\theta _3-1) ((d_{12})_*-\epsilon )=\lambda _3,\\&\theta _3 (r_{2e}-\epsilon )+(1-\theta _3)d_{21}=\theta _3\lambda _3, \end{aligned}\right. \end{aligned}$$

further,

34 $$\begin{aligned} \left\{ \begin{aligned} \theta _3-1&=\frac{\lambda _3-((r_1)_*-\epsilon )}{(d_{12})_*-\epsilon }>0,\\ 1-\theta _3&=\theta _3\frac{\lambda _3-(r_{2e}-\epsilon )}{d_{21}}<0, \end{aligned}\right. \end{aligned}$$

this implies that $(r_1)_*-\epsilon<\lambda _3<r_{2e}-\epsilon$. Denote $u=\lambda _3-((r_1)_*-\epsilon )>0$, $v=(r_1)_*-r_{2e}<0$. From (34), we have

35 $$\begin{aligned} h(u)=u^2+(v+(d_{12})_*-\epsilon +d_{21})u+((d_{12})_*-\epsilon )v=0, \end{aligned}$$

there exist two real roots to quadratic equation (35),

$$\begin{aligned} u_1=\frac{-(v+d_{21}+(d_{12})_*-\epsilon )+\sqrt{(v+d_{21}-(d_{12})_*+\epsilon )^2+4d_{21}((d_{12})_*-\epsilon )}}{2}>0,\\u_2=\frac{-(v+d_{21}+(d_{12})_*-\epsilon )-\sqrt{(v+d_{21}-(d_{12})_*+\epsilon )^2+4d_{21}((d_{12})_*-\epsilon )}}{2}<0. \end{aligned}$$

Since $h(-v)=-d_{21}v>0$, thus $0<u_1<-v$, further,

$$\begin{aligned} \left\{ \begin{aligned} \theta _3&=\frac{u_1}{(d_{12})_*-\epsilon }+1>1,\\ \lambda _3&=u_1+((r_1)_*-\epsilon )<r_{2e}-\epsilon . \end{aligned}\right. \end{aligned}$$

From (33), we have

36 $$\begin{aligned} \begin{aligned} d(x_1+\theta _3 x_2)&\ge [\lambda _3(x_1+\theta _3 x_2)-\max \{a_1, \frac{a_2}{\theta _3}\}(x_1+\theta _3x_2)^2]dt +\sigma _1(t)x_1dB_1(t)+\theta _3\sigma _2(t)x_2dB_2(t). \end{aligned} \end{aligned}$$

When $((r_1)_*+r_{2e}-d_{21}-(d_{12})_*)+\sqrt{((r_1)_*-r_{2e}+d_{21}-(d_{12})_*)^2+4(d_{12})_*d_{21}}>\hat{\sigma }^2$, we can select an $\epsilon$ small enough such that condition $\lambda _3>0.5\hat{\sigma }^2$ holds. We conclude from (36) that

$$\begin{aligned} \langle x_1(t)+\theta _3x_2(t)\rangle _*\ge \frac{\lambda _3-0.5\hat{\sigma }^2}{\max \{a_1, \frac{a_2}{\theta _3}\}}>0, a.s. \end{aligned}$$

*Case* (*iii*): If $(r_1)_*>r_{2e}$, the following proof is similar to Theorem 3, we omit it.

The proof of Theorem 4 is competed. $\square$

***Remark 5 *** If $\rho =0$, when

$$\begin{aligned} (r_{1})_*+r_{2e}-d_{12}-d_{21}+\sqrt{((r_1)_*-r_{2e}+d_{12}+d_{21})^2+4d_{12}(r_{2e}-(r_{1})_*)}>\hat{\sigma }^2, \end{aligned}$$

the population x is strongly permanent in the mean.

#### Theorem 5

*If* $r_{1e}-\frac{\delta fb}{h(g+m)\gamma }-d_{12}(1+\rho \eta )>0$ *and* $r_{2e}-d_{21}>0$*, we have*

$$\begin{aligned} \langle x_1(t)\rangle _*\ge \frac{r_{1e}-\frac{\delta fb}{h(g+m)\gamma }-d_{12}(1+\rho \eta )}{a_1},~~\langle x_2(t)\rangle _*\ge \frac{r_{2e}-d_{21}}{a_2},~ a.s. \end{aligned}$$

#### *Proof*

From (4), we have

$$\begin{aligned} \begin{aligned}{}&dx_1\ge x_1\left[ r_{1e}+(r_{10}-r_{1e})e^{-\mu _1 t}-\delta c_o(t)-d_{12}\left( 1+\frac{\rho c_e^2(t)}{1+\alpha c_e^2(t)}\right) -a_1x_1\right] dt+\sigma _1(t)x_1dB_1(t),\\&dx_2\ge x_2[r_{2e}+(r_{20}-r_{2e})e^{-\mu _2 t}-d_{21}-a_2x_2]dt+\sigma _2(t)x_2dB_2(t).\\ \end{aligned} \end{aligned}$$

Then,

37 $$\begin{aligned} \frac{\ln x_1(t)-\ln x_1(0)}{t}\ge & {} t^{-1}\int _0^t r_1(s)-d_{12}(s)ds-a_1 t^{-1}\int _0^t x_1(s)ds+t^{-1}\int _0^t\sigma _1(s)dB_1(s), \end{aligned}$$

38 $$\begin{aligned} \frac{\ln x_2(t)-\ln x_2(0)}{t}\ge & {} t^{-1}\int _0^t r_2(s)-d_{21}ds-a_2 t^{-1}\int _0^t x_2(s)ds+t^{-1}\int _0^t\sigma _2(s)dB_2(s). \end{aligned}$$

According to Lemma 4, (37) and (38), we obtain that

$$\begin{aligned} \langle x_1(t)\rangle _*\ge \frac{r_{1e}-\frac{\delta fb}{h(g+m)\gamma }-d_{12}(1+\rho \eta )}{a_1},~~\langle x_2(t)\rangle _*\ge \frac{r_{2e}-d_{21}}{a_2},~ a.s. \end{aligned}$$

This ends the proof of Theorem 5. $\square$

### Stochastic single-species population model for migration between two non-polluted patches

If there is no polluted patch, the model (3) will degenerate into the following stochastic single-species population migration model.

39 $$\begin{aligned} \left\{ \begin{aligned} dx_1(t)&=\left[ x_1(t)(r_{1e}+(r_{10}-r_{1e})e^{-\mu _1t}-a_1x_1(t))+d_{21}x_2(t)-d_{12}x_1(t)\right] dt+\sigma _1(t)x_1(t)dB_1(t),\\ dx_2(t)&=\left[ x_2(t)(r_{2e}+(r_{20}-r_{2e})e^{-\mu _2 t}-a_2x_2(t))+d_{12}x_1(t)-d_{21}x_2(t)\right] dt+\sigma _2(t)x_2(t)dB_2(t).\\ \end{aligned}\right. \end{aligned}$$

From Theorems 3 and 4, we can also get the following results for system (39).

#### Corollary 3.1

(i) *Species in system* (39) *will be extinct if*

$$\begin{aligned} r_{1e}+r_{2e}-d_{12}-d_{21}+\sqrt{(r_{1e}-r_{2e}+d_{21}-d_{12})^2+4d_{12}d_{21}}<\sigma ^2. \end{aligned}$$

(ii) Species in system (39) is permanent in the mean if

$$\begin{aligned} r_{1e}+r_{2e}-d_{12}-d_{21}+\sqrt{(r_{1e}-r_{2e}+d_{21}-d_{12})^2+4d_{12}d_{21}}>\hat{\sigma }^2. \end{aligned}$$

## Numerical simulation and discussions

In this section, we give some numerical simulations to demonstrate the analytical results for the SDE model (3) presented in the previous sections by applying the positive preserving truncated Euler-Maruyama method (PPTEMM) given in^32,33^.

**Figure 1:**
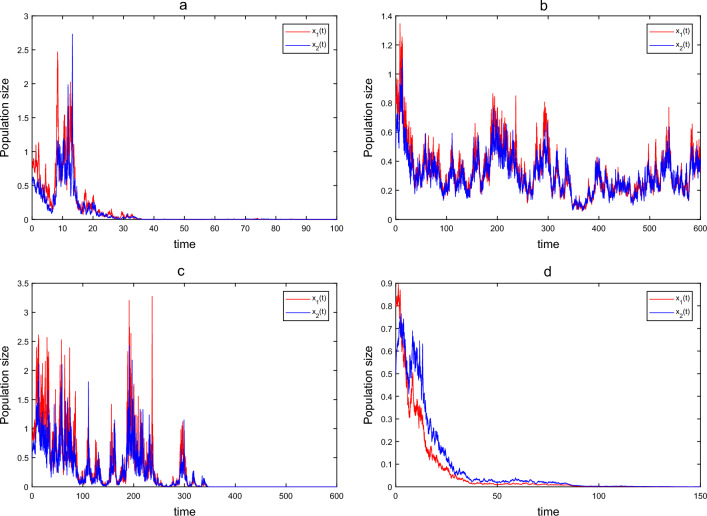
Time series of SDE model (3) with parameters given in (40) for different $\mu _i,~\xi _i, \gamma ,~b$. (a): $\mu _1=0.1$, $\xi _1=0.4$, $\mu _2=0.1$, $\xi _2=0.4$, $\gamma =1$, $b=0.1$; (b): $\mu _1=0.1$, $\xi _1=0.1$, $\mu _2=0.1$, $\xi _2=0.1$, $\gamma =1$, $b=0.1$; (c): $\mu _1=0.01$, $\xi _1=0.1$, $\mu _2=0.01$, $\xi _2=0.1$, $\gamma =1$, $b=0.1$; (d): $\mu _1=0.1$, $\xi _1=0.1$, $\mu _2=0.1$, $\xi _2=0.1$, $\gamma =0.8$, $b=0.2$.

We give some parameters as:

40 $$\begin{aligned} \begin{aligned}{}&r_{1e}=0.3, r_{10}=0.6, \delta =0.8, a_1=0.1, r_{2e}=0.15, r_{20}=0.3, a_2=0.5, d_{12}=0.5, d_{21}=0.6, \\&\alpha =0.2, h=0.3, \rho =1.2, f=0.5, g=0.3, m=0.2, \end{aligned} \end{aligned}$$

and initial value $(x_1(0), x_2(0), c_o(0), c_e(0))=(0.8, 0.5, 0.2, 0.6)$. And then we take different values of $\xi _i$, $\mu _i$, $\gamma$ and *b* to show the influence of the intensity of white noise $\xi _i$, the speed of reversion $\mu _i$, the pulse input cycle of toxicant $\gamma$ and the toxicant input amount each time *b* on the dynamics of the SDE model (3). We first take $\mu _1=0.1$, $\mu _2=0.1$, $\gamma =1$, $b=0.1$. If we choose $\xi _1=0.4$ and $\xi _2=0.4$, by calculation, we have $r_1^*-r_{2e}<-0.1132<0$ and $(r_1^*+r_{2e}-d_{21}-(d_{12})_*)+\sqrt{(r_1^*-r_{2e}+d_{21}-(d_{12})_*)^2+4(d_{12})_*d_{21}}-\sigma ^2<-0.2091<0$, which satisfy condition (ii) in Theorem 3. From Theorem 3, we can obtain that species will be extinct as shown in Fig. 1a. If we choose $\xi _1=0.1$, $\xi _2=0.1$, after calculating, we obtain that $(r_1)_*-r_{2e}<0$ and

$$\begin{aligned} (r_1)_*+r_{2e}-(d_{12})_*-d^*_{21}+\sqrt{((r_1)_*-r_{2e}-(d_{12})_*+d_{21})^2+4(d_{12})_*d_{21}}-\hat{\sigma }^2>0.1323>0, \end{aligned}$$

it implies by Theorem 4 that system (3) is strongly permanent in the mean, see Fig. 1b. We can easily find that higher intensity of white noise $\xi _i$ may lead to the extinction of species by comparing Fig. 1a,b. In the following, we will show the influence of speed of reversion $\mu _i~(i=1, 2)$ on the population dynamics of SDE model (3). We take $\mu _1=0.01$, $\xi _1=0.1$, $\mu _2=0.01$, $\xi _2=0.1$, $\gamma =1$, $b=0.1$, and derive that $(r_1^*+r_{2e}-d_{21}-(d_{12})_*)+\sqrt{(r_1^*-r_{2e}+d_{21}-(d_{12})_*)^2+4(d_{12})_{*}d_{21}}-\sigma ^2<-0.0591<0$. It follows from Theorem 3 that the single-species dies out (see Fig. 1c). From Fig. 1b,c, we can find that a small speed of reversion $\mu _i$ can give rise to extinction of species. Finally, we will show the influence of $\gamma$ and *b* on species survival, and take $\mu _1=0.1$, $\xi _1=0.1$, $\mu _2=0.1$, $\xi _2=0.1$, $\gamma =0.8$, $b=0.2$, simple calculation obtain that $r_1^*-r_{2e}=-0.5112<0$ and $(r_1^*+r_{2e}-d_{21}-(d_{12})_*)+\sqrt{(r_1^*-r_{2e}+d_{21}-(d_{12})_*)^2+4(d_{12})_*d_{21}}-\sigma ^2=-0.0482<0$, which satisfy the condition (ii) of the Theorem 3, hence, population $x_i$ goes to extinction (see Fig. 1d). Comparing Fig. 1b,d, we can observe that species may tend to survive when increasing the toxins input period $\gamma$ or decreasing the toxins input amount *b*.

**Figure 2:**
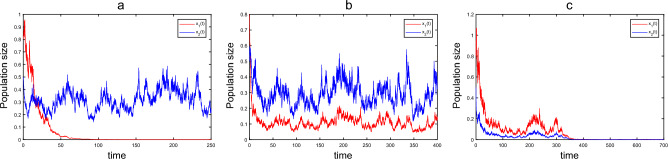
Time series of SDE model (3) with parameters given in (41) for different migration rate. (**a**) $d_{12}=d_{21}=0$; (**b**) $d_{12}=0.4$, $d_{21}=0.2$, $\rho =1.2$; (**c**) $d_{12}=0.2$, $d_{21}=0.8$, $\rho =0.4$.

On the other hand, population migration both patches will also affect the survival of the single-species. We choose parameters as

41 $$\begin{aligned} \begin{aligned}{}&r_{1e}=0.25, r_{10}=0.4, \delta =0.8, a_1=0.1, r_{2e}=0.2, r_{20}=0.1, a_2=0.6, \alpha =0.1, \\&h=0.3, \gamma =1, b=0.12, \xi _1=0.1, \mu _1=0.2, \xi _2=0.1, \mu _2=0.2, \end{aligned} \end{aligned}$$

and initial value $(x_1(0), x_2(0), c_o(0), c_e(0))=(0.8, 0.5, 0.2, 0.6)$. If $d_{12}=d_{21}=0$, that is, there is no mutual migration between populations of two patches. According to the theoretical results of Ref.^21^, we know that population $x_i$ is permanent in the mean if $\langle r_i(t)-0.5\sigma _i(t)\rangle >0$, and population $x_i$ goes to extinction if $\langle r_i(t)-0.5\sigma _i(t)\rangle <0$. After calculation, we have $\langle r_1(t)-0.5\sigma _1(t)\rangle =r_{1e}-\frac{\delta b}{h\gamma }-\frac{1}{2}\frac{\xi _1^2}{2\mu _1}=-0.0825<0$ and $\langle r_2(t)-0.5\sigma _2(t)\rangle =r_{2e}-\frac{1}{2}\frac{\xi _2^2}{2\mu _2}=0.1875>0$, population $x_1$ in patch 1 will be extinct and population $x_2$ in patch 2 is strongly persistent in the mean, see Fig. 2a. Moreover, when we take $d_{12}=0.4$, $d_{21}=0.2$, $\rho =1.2$, and calculate that $(r_1)_*-r_{2e}=-0.2722<0$ and $(r_1)_*+r_{2e}-(d_{12})_*-d_{21}+\sqrt{((r_1)_*-r_{2e}-(d_{12})_*+d_{21})^2+4(d_{12})_*d_{21}}-\hat{\sigma }^2>0.2492>0$. According to Theorem 3’s (ii), we know that species is strongly permanent in the mean, as shown in Fig. 2b. However, If we take $d_{12}=0.2$, $d_{21}=0.8$, $\rho =0.4$, it can be calculated that $(r_1)_*-r_{2e}=-0.2659<0$ and $(r_1)_*+r_{2e}-(d_{12})_*-d_{21}+\sqrt{((r_1)_*-r_{2e}-(d_{12})_*+d_{21})^2+4(d_{12})_*d_{21}}-\hat{\sigma }^2<-0.0039<0$ by Theorem 3’s (ii), which means species goes extinct, see Fig. 2c.

## Conclusions

With the rapid growth of economy, a large number of toxic substances are discharged into the ecosystem, which seriously threatens the survival of species and human beings. Based on its theoretical and practical significance, stochastic population models with impulsive toxicant input and stochastic single-species population models with migration have attracted many scholars’ attention (see, e.g.,^14–31^). Up to our knowledge, few studies have considered the influence of environmental toxins on population migration between patches. In this paper, we propose and study a stochastic single-species population system with migration driven by environmental toxicant and impulsive toxicant input. We prove the existence and uniqueness of the global positive solution of SDE (3) by constructing the Lyapunov function, and analyze the boundedness of the p-moments of the solution. And then, we obtain sufficient conditions for population extinction, stochastic permanence and permanence in the mean. There results show that the intensity of white noise $\xi _i$, the speed of reversion $\mu _i$, the pulse input period of toxicant $\gamma$, the toxicant input amount each time *b* and the population migration between patches play a very important role on the survival of the population, see Figs. 1 and 2. Finally, we also study the stochastic single-species population model with migration between two non-polluted patches, and give the sufficient conditions for population extinction and permanence.

On the other hand, there are many interesting problems that deserve further study, for example, the existence and uniqueness of the ergodic stationary probability density for system (3) (see^34,35^), and many more realistic but complex models should be formulated (see^36^). In addition, the telegraph noise can be illustrated as a switching between two or more regimes of environment, which differ by factors such as nutrition or as rain falls^37,38^, which is memoryless and the waiting time for the next switch has an exponential distribution, we can use a finite-state Markov chain to simulate regime switching in here. Therefore, it is interesting to introduce the telegraph noise into model (3). We shall also consider this question in our future work.

## Data Availability

All data used in this study have been given within the article.
